# Complement-independent bystander injury in AQP4-IgG seropositive neuromyelitis optica produced by antibody-dependent cellular cytotoxicity

**DOI:** 10.1186/s40478-019-0766-7

**Published:** 2019-07-11

**Authors:** Tianjiao Duan, Alex J. Smith, Alan S. Verkman

**Affiliations:** 1 Departments of Medicine and Physiology, University of California, 1246 Health Sciences East Tower, 513 Parnassus Ave, San Francisco, CA 94143-0521 USA; 2 Department of Neurology, Second Xiangya Hospital of Central South University, Changsha, 410011 Hunan China

**Keywords:** NMO, Aquaporin-4, ADCC, Leukocyte, Astrocyte

## Abstract

Cellular injury in AQP4-IgG seropositive neuromyelitis spectrum disorder (herein called NMO) involves AQP4-IgG binding to astrocytes, resulting in astrocyte injury by complement-dependent cytotoxicity (CDC) and antibody-dependent cellular cytotoxicity (ADCC) mechanisms. The rapid disease progression, severe tissue damage, and abundant leukocyte infiltration seen in some NMO patients suggest a more direct mechanism for demyelination and neurologic deficit than secondary injury from astrocyte loss. Here, we report evidence for an ‘ADCC bystander mechanism’ in NMO involving injury to nearby cells by leukocytes following their activation by AQP4-bound AQP4-IgG on astrocytes. In model cocultures containing AQP4-expressing and null CHO cells, AQP4-IgG and complement killed bystander null cells to ~ 100 μm away from AQP4-expressing cells; AQP4-IgG and NK cells produced bystander killing to ~ 300 μm, with perforin deposition seen on injured null cells. Bystander cytotoxicity was also seen with neutrophil-mediated ADCC and in astrocyte-neuron cocultures. Mechanistic studies, including real-time imaging, suggested that leukocytes activated by an AQP4-dependent ADCC mechanism injure bystander cells by direct targeted exocytosis on neighboring cells and not by diffusion of soluble granule contents. In support of this conclusion, ADCC bystander injury was preferentially reduced by an RGDS peptide that inhibits integrin adhesion. Evidence for ADCC bystander injury to oligodendrocytes and neurons was also found in mice following intracerebral injection of AQP4-IgG and NK cells, which was inhibited by RGDS peptide. These results establish a novel cellular pathogenesis mechanism in AQP4-IgG seropositive NMO and provide evidence that inflammatory mechanisms can cause widespread tissue damage in NMO independently of the secondary effects from astrocyte loss.

**Electronic supplementary material:**

The online version of this article (10.1186/s40478-019-0766-7) contains supplementary material, which is available to authorized users.

## Introduction

Neuromyelitis optica spectrum disorder (NMOSD) is an inflammatory demyelinating disease of the central nervous system distinct from multiple sclerosis. Most NMOSD patients are seropositive for IgG1 autoantibodies against aquaporin-4 (AQP4) [31, 32], a plasma membrane water channel expressed on astrocytes but not on other cell types in the central nervous system [21, 39, 42]. An initiating event in seropositive NMOSD (herein called NMO) is binding of anti-AQP4 autoantibodies (called AQP4-IgG) to AQP4 on astrocytes [27], which causes direct astrocyte injury by complement-dependent cytotoxicity (CDC) [23, 49, 54] and antibody-dependent cellular cytotoxicity (ADCC) [10, 45, 47, 63] mechanisms. Injury to surrounding non-AQP4-expressing ‘bystander’ cells, such as oligodendrocytes, neurons and endothelial cells, leads to demyelination, neuron loss and consequent neurological deficit, which can include visual and motor deficits. It has been suggested that tissue damage in NMO is a secondary consequence of astrocyte loss [24, 27, 41], though inflammatory mechanisms may directly damage surrounding tissue in antibody-mediated autoimmunity [45].

Injury to astrocytes by a CDC mechanism involves AQP4-IgG binding to AQP4 followed by binding of complement protein C1q and activation of the classical complement pathway, resulting in the generation of anaphylatoxins and membrane attack complex (MAC) [4, 41, 52]. We recently reported a ‘complement bystander injury’ mechanism in NMO in which bystander cells near astrocytes, including oligodendrocytes, neurons and perhaps other cells, are injured following complement activation on astrocytes by local diffusion of short-lived, activated complement components leading to MAC formation on bystander cells [19, 60]. We proposed that complement bystander injury may contribute to the early and marked demyelination and neuronal injury seen in human NMO and experimental animal models of NMO.

Astrocyte injury by an ADCC mechanism in NMO involves AQP4-IgG binding to astrocytes followed by binding and activation of various leukocytes, such as granulocytes, macrophages or NK cells, via Fcγ receptors [4, 34, 47, 68]. ADCC-mediated astrocyte injury can occur by a variety of mechanisms, including release of toxic granule contents such as perforin and proteases [53, 67]. Evidence for ADCC in NMO pathogenesis comes from human pathology showing prominent leukocyte infiltration and activation [33, 37, 49], in vitro cell models [10, 63], and experimental animal models [47, 68].

Leukocyte infiltration is associated with severe, necrotic NMO lesions [38]. Here, we postulated that an analogous ‘ADCC bystander injury’ mechanism could damage non-AQP4-expressing cells near astrocytes following Fcγ receptor-mediated leukocyte activation. We report evidence, using coculture systems and mice, that activation of NK cells or neutrophils by AQP4-IgG-coated AQP4 expressing cells can lead to acute lysis of non-AQP4 expressing neighboring cells, supporting ADCC bystander injury as a mechanism of tissue damage in NMO.

## Materials and methods

### Materials

Purified human monoclonal recombinant AQP4-IgG (rAb-53) [10, 16] was provided by Dr. Jeffrey Bennett (University of Colorado, Aurora, CO). Sera from confirmed seropositive NMO patients was provided by the Circles Bloodbank of the Guthy-Jackson Charitable Foundation. Human complement was purchased from Innovative Research (Novi, MI) and human (control) IgG from Thermo Fisher Scientific (Rockford, IL). Sprague-Dawley rats were purchased from Charles River Laboratories (Wilmington, MA) and bred at UCSF. All animal procedures were approved by the University of California, San Francisco Animal Care and Use Committee (IACUC).

### Cell culture

Chinese hamster ovary (CHO) cells stably expressing human AQP4-M23 (named CHO-AQP4 cells), as described [16, 44], were cultured at 37 °C in 5% CO_2_ 95% air in F-12 Ham’s Nutrient Mixture medium supplemented with 10% fetal bovine serum, 200 μg/ml geneticin, 100 U/ml penicillin and 100 μg/ml streptomycin. Non-transfected CHO-K1 cells without AQP4 (called CHO-null cells) were cultured in the same medium but without geneticin. Human natural killer cells (NK cells) transfected to express the high-affinity 176 V variant of the Fcγ receptor IIIA [66] were obtained from Fox Chase Cancer Center (Philadelphia, PA). NK cells were cultured in suspension in α-MEM (Sigma-Aldrich, St. Louis, MO) containing 0.1 mM 2-mercaptoethanol, 2 mM L-glutamine, 0.2 mM myoinositol, 10% FBS, 10% horse serum, 2.5 μM folic acid, 5 ml non-essential amino acids, 1 mM Na pyruvate, 1% penicillin/streptomycin, and 100 IU/ml of human recombinant interleukin-2 (GenScript, Piscataway, NJ). Primary astrocyte-neuron cocultures were generated from brains of embryonic day 18 Sprague-Dawley rats (neurons) or cerebral cortex of neonatal rats (astrocytes) as described [19].

Neutrophils were isolated from human peripheral blood by a modified Ficoll-Hypaque method [20, 40] using density gradient centrifugation and a commercial separation medium (Lympholyte-poly, CL5071, Cedarlane Labs, Burlington, NC) containing sodium metrizoate and dextran 500. After separation, red cell lysis buffer was added to the neutrophil layer to lyse residual red cells. The isolated neutrophils were resuspended in HBSS/HSA solution (2% HSA) at specified concentration and used within 2 h. Neutrophil purity was 97.0% as judged by immunofluorescence using neutrophil-specific CD66b antibody (1:200, 555723, BD Pharmingen, San Jose, CA).

### Complement-dependent cytotoxicity

CHO-AQP4 and CHO-null cells were cultured individually or at specified cell number ratios (from 1:5 to 1:50) on coverslips in 24-well plates, and grown for 18-24 h until confluent. For assay of CDC, CHO cells were incubated for 2 h at 37 °C with 5% human complement and 10 μg/ml AQP4-IgG (or control human IgG) in Hank’s balanced salt solution (HBSS, pH 7.2; Invitrogen, Camarillo, CA).

### Antibody-dependent cellular cytotoxicity

For assay of ADCC, CHO-AQP4 and CHO-null cell cocultures were generated as for CDC experiments. Primary neurons were grown on coverslips in 12-well plates and astrocytes were added to neurons at a 1:20 ratio before experiments. NK-92 cells overexpressing CD16 were used as the effector cells. Cells were pre-incubated with 5 μg/ml AQP4-IgG (or control IgG) for 30 min at 37 °C. In some experiments, cells preincubated with a 1:100 dilution of serum of an AQP4-IgG seropositive NMO patient. NK cells or neutrophils were then added to the AQP4-IgG-coated CHO cells at an effector: target cell ratio of 5:1, and incubated for an additional 1 h at 37 °C. Following treatments, cells were washed extensively with Hank’s Balanced Salt Solution (HBSS) to remove the remaining effector cells. In some studies, NK cells were pretreated with an inhibitor of the perforin cytotoxic pathway, concanamycin A (CMA, 10 nM, C9705, Sigma-Aldrich) [66] for 2 h prior to AQP4-IgG addition. In some studies, a neutralizing anti-perforin antibody (δG9, 10 μg/ml, BD Pharmingen, San Jose, CA) was added together with NK cells onto the CHO cells. In some studies, the RGD-containing peptide RGDS (or control peptide RGES) (200 μM, Sigma-Aldrich) [28] was pre-incubated with CHO cell cocultures for 1 h prior to AQP4-IgG addition.

### Immunofluorescence

In some experiments, a fixable red-fluorescent dead-cell stain (L23102, amine-reactive dye, Invitrogen, Eugene, OR) at 1:1000 dilution was added 30 min prior to cell fixation. Cells were then rinsed in PBS and fixed with 4% paraformaldehyde (PFA) for 15 min. After fixation, cells were blocked for 1 h with 1% BSA and 0.1% Triton-X100 in PBS, and incubated with primary antibodies. Brains were cut as 7-μm-thick frozen sections, and blocked in the same buffer before immunostaining. Cultures and brain sections were incubated at 4 °C overnight with antibodies against AQP4 (sc-20812, 1:200, Santa Cruz Biotechnology, Dallas, TX), AQP4 (sc-9888, 1:200, Santa Cruz Biotechnology, Dallas, TX), C1q (ab71940, 1:50, abcam, Cambridge, MA), C5b-9 (ab55811, 1:200, abcam, Cambridge, MA), GFAP (AB5541, 1:1000; Millipore, Burlington, MA), MAP2 (PA5–17646, 1:100, Thermo Fisher Scientific, Rockford, IL), perforin (δG9, 556434, 1:100, BD Pharmingen, San Jose, CA), NeuN (ABN78, 1:200, Millipore, Burlington, MA), Olig2 (sc-48817, 1:100, Santa Cruz Biotechnology, Dallas, TX) or GFP (MA1–952, 1:20, Thermo Fisher Scientific, Rockford, IL) followed by 1 h incubation with appropriate species-specific Alexa Fluor-conjugated secondary antibody (5 μg/ml each, Invitrogen, Camarillo, CA). Sections and coverslips were mounted with ProLong Gold antifade reagent (P36931, Thermo Fisher Scientific, Rockford, IL), and immunofluorescence was visualized on a Nikon confocal microscope using a 20x/0.5 N.A., 60x/1.25 N.A., or 100x/1.4 N.A. oil objective lens.

### Real-time imaging

For live-cell real-time imaging, CHO-AQP4 / CHO-null cell cocultures were imaged using a 20x, 0.45 NA objective lens on a Nikon Eclipse Ti microscope equipped with an environmental chamber at 37 °C and 5% CO_2_. CHO-AQP4 cells were loaded with the CellTracker™ Green CMFDA (C2925, 5 μM in HBSS) for 30 min at 37 °C, cell tracker was removed and cells were replated with unlabeled CHO-null cells overnight before experiments. Control experiments verified that 100% of CHO-AQP4 cells retained the CellTracker label overnight. Cells were then pre-incubated with 5 μg/ml AQP4-IgG for 30 min, which was then washed away prior to imaging. For imaging, cells were then incubated in HBSS containing ethidium homodimer-1 (1 μM, Invitrogen, Eugene, OR). Transmitted light (phase-contrast), green and red fluorescence images were obtained sequentially every 2 min for a 30 min baseline period and then for 1.5 h following addition of NK cells.

### Mouse studies

AQP4 knockout (AQP4^−/−^) mice used in this study have been described and extensively characterized [35]. Experiments were done on weight-matched wild type and AQP4^−/−^ mice on a CD1 genetic background, generally of age 16–18 weeks. Mice were anesthetized with ketamine (100 mg/kg) and xylazine (10 mg/kg) and mounted on a stereotaxic frame. A midline scalp incision was made and a burr hole of diameter 1 mm was drilled in the skull 0.5 mm anterior and 2 mm lateral to the bregma. A glass pipette with a 40-μm-diameter tip was inserted 3 mm deep to infuse 4 μg AQP4-IgG (or control IgG) together with 10^4^ GFP labeled NK-cells and 3 μM dead cell dye ethidium homodimer (EH-1) at ~ 1 μl/min for a total volume of 3 μl. In some experiments, AQP4-IgG and NK cells were injected together with RGDS peptide or RGES peptide (1 mM, 2 μl). After 90 min, mice were anesthetized and perfused through the left cardiac ventricle with 100 ml PBS and then 100 ml of 4% PFA in PBS. Brain were post-fixed overnight in 4% PFA and cryoprotected in 20% sucrose for cutting 7-μm-thick sections on a cryostat.

### Image analysis

To quantify the fraction of dead cells surrounding AQP4 expressing cells, low magnification (5x) images were used. ImageJ software was used to draw concentric circles of 100 μm, 200 μm and 300 μm radius around single, isolated AQP4-expressing cells. Dead and total cells within each circle were counted from the red fluorescence and transmitted light images, respectively.

### Statistics

Data are presented as mean ± S.E.M. Statistical analysis and graphing were performed with GraphPad Prism 6 software. Statistical comparisons were made using the two-way ANOVA for comparisons within a group data set.

## Results

### Complement bystander cytotoxicity in CHO cell cocultures

**Fig. 1:**
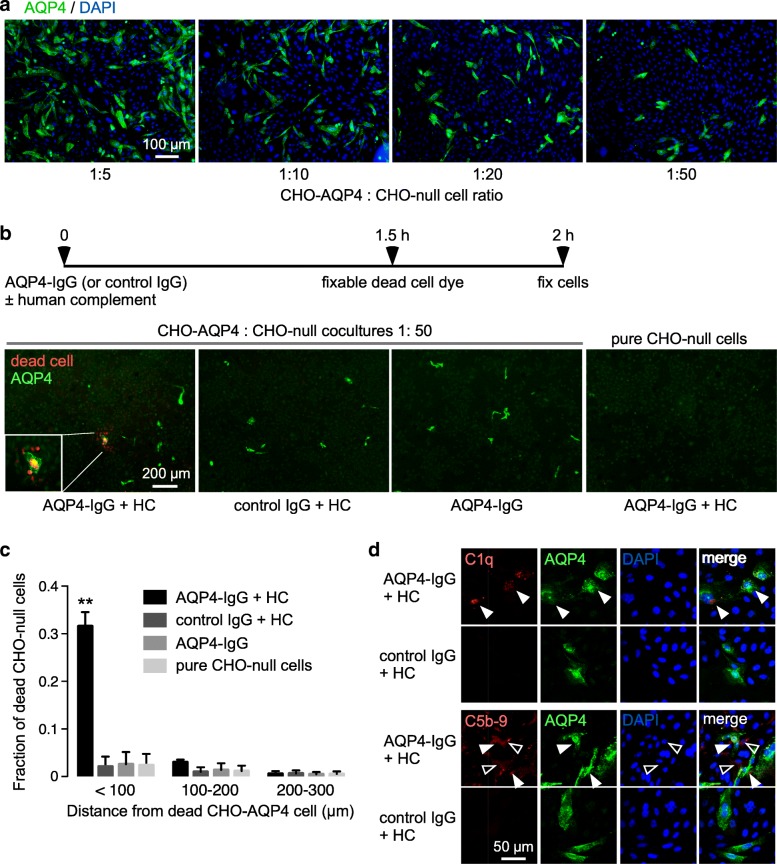
Complement bystander cytotoxicity in cocultures containing AQP4-expressing and null CHO cells. **a** AQP4 immunofluorescence (green) with DAPI counterstain (blue) in cocultures of CHO-AQP4 and CHO-null cells plated at different cell ratios. **b** Complement-dependent cytotoxicity. Cocultures at 1:50 cell ratio was incubated with 10 μg/ml AQP4-IgG (or control IgG) and 5% human complement (HC), with fixable red-fluorescent dead cell marker (left). AQP4 immunofluorescence (green) with dead cell stain (red) for cells incubated with AQP4-IgG and HC, with controls including cells incubated with control IgG and HC, with AQP4-IgG alone, and pure CHO-null cells incubated with AQP4-IgG and HC (right). **c** Fraction of red-stained dead CHO-null cells as a function of distance from dead CHO-AQP4 cells (mean ± S.E.M., 3 slides with > 50 dead cells analyzed, ** *P*<0.01 comparing AQP4-IgG + HC vs. control IgG + HC or AQP4-IgG or pure CHO-null cells by two-way ANOVA). **d** C1q (top) and C5b-9 (bottom) immunofluorescence (red) in cocultures incubated as in (**b)**, costained with AQP4 (green) and DAPI (blue). White filled arrows indicate C5b-9 or C1q on CHO-AQP4 cells, white open arrows show C5b-9 on CHO-null cells

A CHO cell coculture system was established to study bystander cytotoxicity mechanisms. CHO cells were chosen for initial model studies because of their rapid growth on uncoated plastic or glass, strong plasma membrane AQP4 expression following transfection, and ability to generate cultures with well-isolated AQP4-expressing cells surrounded by bystander cells. Figure 1a shows AQP4 immunofluorescence, with DAPI counterstain, of cocultures containing AQP4-expressing CHO cells (CHO-AQP4) and non-transfected CHO cells (CHO-null) at different cell number ratios. At lower ratios, the CHO-AQP4 cells were generally well-separated and surrounded by CHO-null cells. Occasionally, two adjacent CHO-AQP4 cells were seen as a consequence of cell division that occurred between the time of plating and experiment.

A CHO-AQP4 to CHO-null cell ratio of 1:50 was used to study complement bystander cytotoxicity, which was done initially as a reference to compare with ADCC bystander experiments. To study complement bystander cytotoxicity, cocultures were incubated with AQP4-IgG and human complement (HC) for 2 h with a fixable dead cell dye added at 1.5 h (Fig. 1b, top). Figure 1b (bottom, left panel) shows several red-stained dead CHO-null cells surrounding a dead CHO-AQP4 cell. Dead cells were not seen with control (non-NMO) IgG in place of AQP4-IgG, with AQP4-IgG but without HC, and in CHO-null cells exposed to AQP4-IgG and HC (Figs. 1b, 3 right panels). Figure 1c summarizes the analysis of images from 3 cocultures in which the fraction of dead CHO-null cells was determined at different distances from dead CHO-AQP4 cells. Essentially all dead bystander CHO-null cells were seen within 100 μm of dead CHO-AQP4 cells. Time-lapse imaging shows both CHO-AQP4 cells (labeled with green cell tracker) and nearby CHO-null cells were injured, with EH-1 uptake following AQP4-IgG and human complement addition (see Additional file 1: Video S1).

Figure 1d shows immunofluorescence of C1q, an early complement protein that binds to the Fc portion of AQP4-IgG and activates the classical complement pathway, and C5b-9, the terminal membrane attack complex (MAC) that causes cytotoxicity. When cells were exposed to AQP4-IgG and human complement, C1q immunofluorescence was seen only on AQP4-expressing CHO cells and not on CHO-null cells (as stained with DAPI), whereas C5b-9 immunofluorescence was seen on both CHO-AQP4 cells and nearby CHO-null cells. These results, utilizing a simple model system, support our prior data on complement bystander cytotoxicity obtained in astrocyte-oligodendrocyte and astrocyte-neuron cocultures [19, 60].

### ADCC bystander killing in CHO cell cocultures

**Fig. 2:**
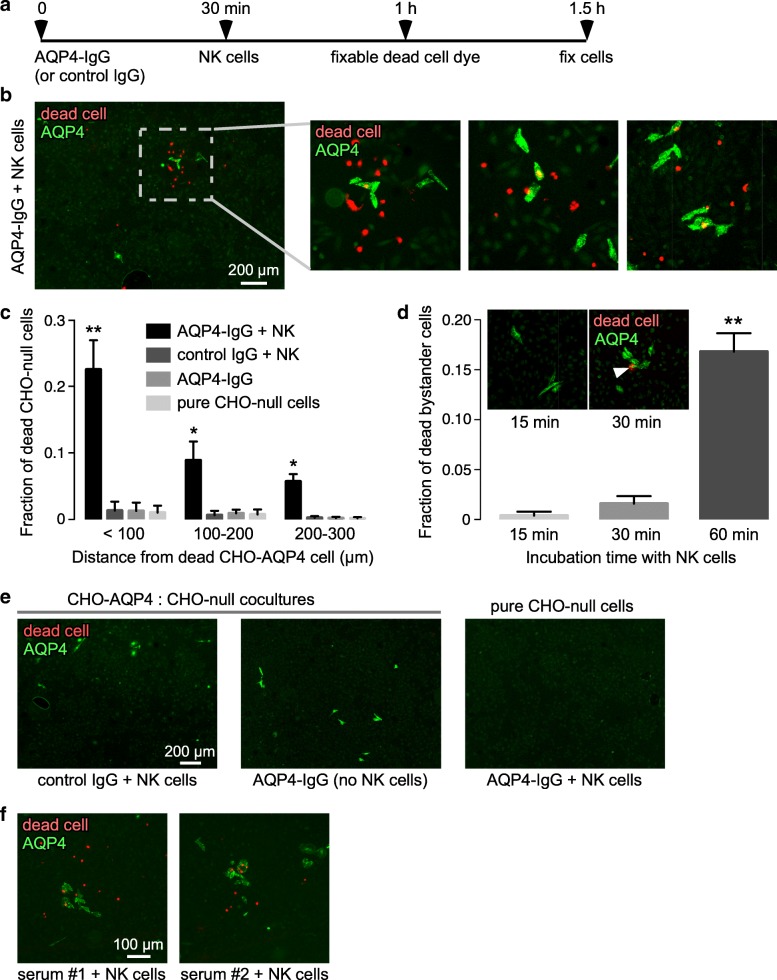
ADCC bystander killing in cocultures containing AQP4-expressing and null CHO cells. **a** Cocultures at 1:50 CHO-AQP4:CHO-null cell ratio were pre-incubated for 30 min with AQP4-IgG (or control IgG) then washed followed by addition of NK cells with a fixable dead cell stain at 30 min prior to fixation. **b** AQP4 immunofluorescence (green) with dead cell stain (red) at low magnification (left) in cocultures incubated with 5 μg/ml AQP4-IgG and NK cells at an effector:target cell ratio of 5:1. Three fields at high magnification are shown (right). **c** Fraction of red-stained, dead CHO-null cells as a function of distance from dead CHO-AQP4 cells (mean ± S.E.M., 5 slides with > 80 dead cells analyzed, ** *P*<0.01, *P<0.05 comparing AQP4-IgG + NK vs. control IgG + NK or AQP4-IgG or pure CHO-null cells by two-way ANOVA). **d** Fraction of dead bystander cells, as in (**c**), as a function of incubation time with NK cells (mean ± S.E.M., 5 slides, ** *P*<0.01 by unpaired t test). Inset shows representative fields at 15 min and 30 min. White filled arrow indicates dead CHO-AQP4 cell at 30 min. **e** Control studies including CHO-AQP4 and CHO-null cocultures incubated with 5 μg/ml control IgG and NK cells at an effector:target cell ratio of 5:1, with AQP4-IgG alone, and pure CHO-null cells incubated with AQP4-IgG and NK cells. **f** Cocultures were incubated with NK cells and 1% serum from two seropositive NMO patients, and immunostained as in panel (**b**)

The CHO cell coculture model was then used to test whether AQP4-IgG with NK cells could produce ADCC bystander killing in the absence of complement. For these experiments, cocultures were initially coated with AQP4-IgG by incubation and washing (Fig. 2a), which minimized non-specific solution-phase activation of NK cells by unbound IgG. Figure 2b shows examples of apparent ADCC bystander killing in which red-stained, dead CHO-null cells were seen preferentially near dead CHO-AQP4 cells. Analysis of experiments done in 5 cocultures showed that killing of bystander CHO-null cells occurred largely with 100 μm of CHO-AQP4 cells, with some dead cells seen at greater distances up to 300 μm (Fig. 2c). Time course studies showed that ADCC bystander killing was observed at 60 min, with little cell killing at 15 min and killing largely restricted to CHO-AQP4 cells at 30 min (Fig. 2d). Few dead CHO cells were seen in control studies done in the cocultures when control (non-AQP4) IgG was used in place of AQP4-IgG or when NK cells were not included, or in pure CHO-null cultures exposed to AQP4-IgG and NK cells (Fig. 2e). Bystander injury to CHO-null cells nearby CHO-AQP4 cells was also seen using serum from two seropositive NMO patients instead of the recombinant AQP4-IgG antibody (Fig. 2f).

**Fig. 3:**
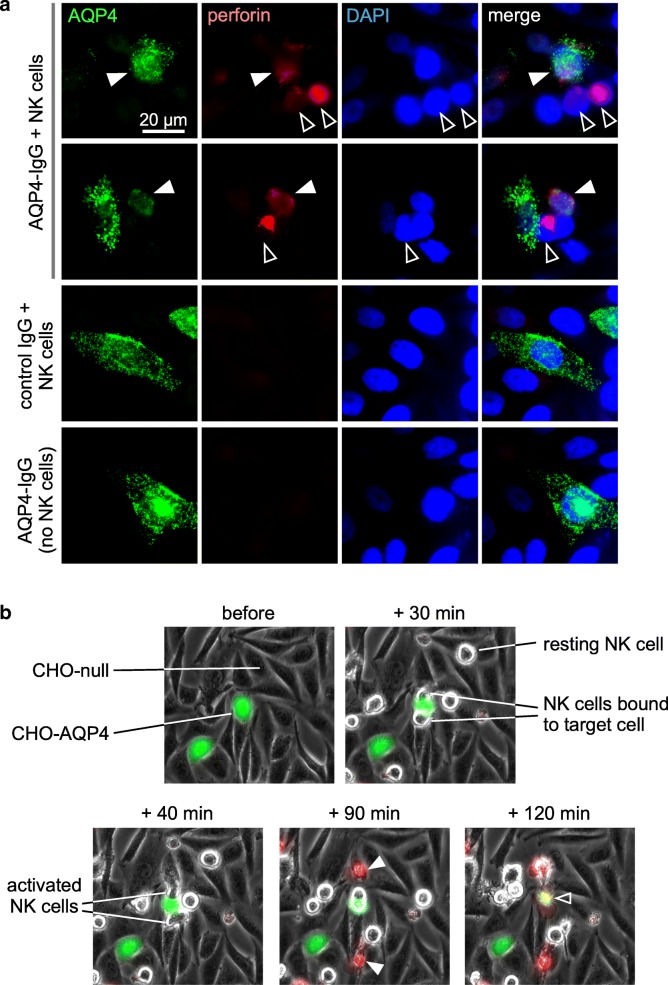
Mechanism of ADCC bystander killing. **a** Perforin immunofluorescence of CHO-AQP4 and CHO-null cocultures after pre-coating with AQP4-IgG and incubation NK cells, as in Fig. 2b, with controls including control IgG and NK cells, and AQP4-IgG alone. White filled arrows indicate perforin on AQP4-expressing CHO cells, white open arrows show perforin on CHO-null cells. **b** Time-lapse imaging of coculture of CHO-AQP4 cells (labeled green with cell tracker) and CHO-null cells, pre-incubated for 30 min AQP4-IgG, then at indicated times after NK cell addition (for explanation see text and Additional file 2: Video S2)


Studies were done to investigate potential mechanisms of ADCC bystander killing by AQP4-IgG and NK cells. In CHO cell cocultures exposed to AQP4-IgG and NK cells, perforin immunostaining was seen on both AQP4-positive CHO cells and AQP4-negative CHO-null bystander cells (Fig. 3a), suggesting the involvement of perforin release in bystander cell killing. Time-lapse imaging was done to visualize the events during ADCC bystander killing. For these studies AQP4-expressing CHO cells were pre-labeled with cell tracker green fluorescent marker (with CHO-null cells not labeled) in order to identify them during real-time imaging. Ethidium homodimer was included, which is rapidly taken up and becomes fluorescent in injured cells. Figure 3b shows images from a time-lapse video (see Additional file 2: Video S2). At 30 min NK cell activation was seen on a green CHO-AQP4 cell, which became stained red at 120 min. An activated NK cell was seen at 40 min to move to nearby bystander cells, which became stained red at 90 min. Examination of > 20 bystander killing events in multiple time-lapse images revealed that in all cases bystander killing involved physical contact between activated NK cells and bystander cells.

**Additional file 2: Video S2** ADCC bystander killing in CHO-AQP4 and CHO-null cell cocultures. Time-lapse imaging of coculture of CHO-AQP4 cells (labeled green with cell tracker) and CHO-null cells, pre-incubated for 30 min with AQP4-IgG, followed by NK cell addition at an effector:target cell ratio of 5:1. Solutions contained dead cell marker EH-1 (red). (AVI 4219 kb)


### ADCC bystander killing in in vitro models of NMO

**Fig. 4:**
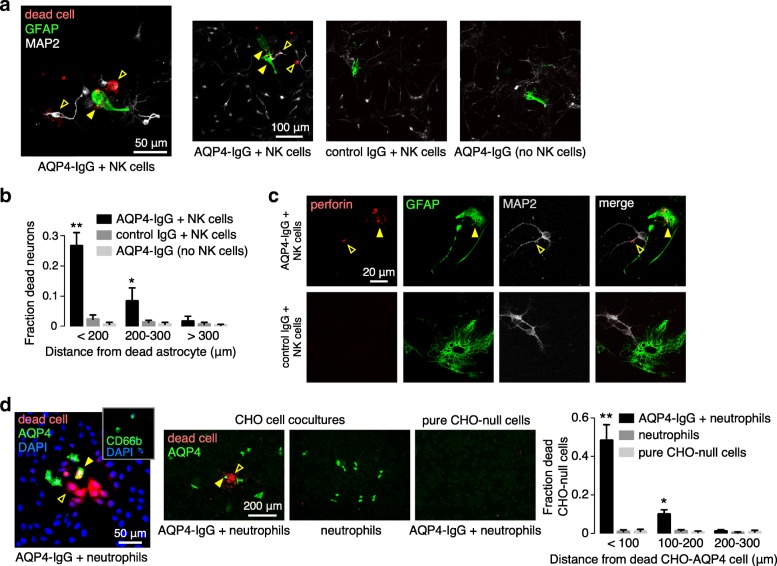
ADCC bystander killing in astrocyte-neuron cocultures. **a** Astrocyte-neuron cocultures following 30 min pre-coating with AQP4-IgG and 2 h incubation with NK cells at an effector:target cell ratio of 5:1, with fixable dead cell marker added for the final 30 min. GFAP (astrocyte) and MAP2 (neuron) immunofluorescence, with dead cells stained red, shown at high (left) and low (right) magnifications. Yellow filled arrows indicate dead astrocytes, yellow open arrows show dead neurons. **b** Fraction of dead neurons at different distances from the center of dead astrocytes (mean ± S.E.M., 3 slides with > 30 dead cells analyzed, ** *P*<0.01, *P<0.05 comparing AQP4-IgG + NK vs. control IgG + NK or AQP4-IgG without NK cells by two-way ANOVA). **c** Perforin immunofluorescence (red) of cocultures treated as in a. with GFAP (green) and MAP2 (gray) immunofluorescence. Yellow filled arrows indicate perforin on astrocytes, yellow open arrows show perforin on neurons. **d** AQP4 immunofluorescence (green) with dead cell stain (red) and DAPI (blue) at high (left) and low (center) magnifications in CHO-AQP4 and CHO-null cocultures following 5 μg/ml AQP4-IgG and neutrophils at an effector:target cell ratio of 5:1. Yellow filled arrows indicate dead CHO-AQP4 cells, yellow open arrows show dead CHO-null cells. (Right) Fraction of dead CHO-null cells at different distances from the center of dead CHO-AQP4 cells (mean ± S.E.M., 3 slides with > 50 dead cells analyzed, ** *P*<0.01, *P<0.05 comparing AQP4-IgG + neutrophils vs. neutrophils or pure CHO-null cells by two-way ANOVA)

To investigate whether ADCC bystander killing, as demonstrated in the AQP4-IgG-exposed CHO coculture / NK cell model system, can occur in cell types that are relevant to NMO neuropathology, experiments were done in primary astrocyte-neuron cocultures in which AQP4 is expressed on astrocytes but not on neurons [19, 42]. Cells were exposed to AQP4-IgG and NK cells for 2 h. Figure 4a shows dead, red-stained MAP2-positive neurons nearby dead GFAP-positive astrocytes. Minimal killing was found in cocultures incubated with control IgG and NK cells or AQP4-IgG without NK cells. Figure 4b summarizes the fraction of dead neurons at different distances from the center of dead astrocytes, showing preferential killing of neurons within 300 μm of dead astrocytes. Perforin deposition was seen on both astrocytes and nearby neurons (Fig. 4c).

ADCC bystander killing studies were also done in the CHO cell coculture system, but using neutrophils instead of NK cells. Freshly isolated human neutrophils were incubated with CHO cell cocultures for 1 h following AQP4-IgG exposure. Figure 4d (left panel) shows bystander killing of CHO-null cells (as stained with DAPI) nearby dead CHO-AQP4 cells (AQP4 stained), with the inset confirming CD66b-positive neutrophil identity. Figure 4d (three center panels) shows that exposure of CHO cell cocultures to neutrophils and AQP4-IgG produces bystander killing by an ADCC mechanism, with killing not seen with neutrophils alone or in pure CHO-null cell cultures. Figure 4d (right panel) summarizes the fraction of dead CHO-null cells at different distances from the center of dead AQP4-expressing CHO cells, showing preferential killing of CHO-null cells within 100 μm of dead CHO-AQP4 cells.

### ADCC bystander killing in a mouse model of NMO

**Fig. 5:**
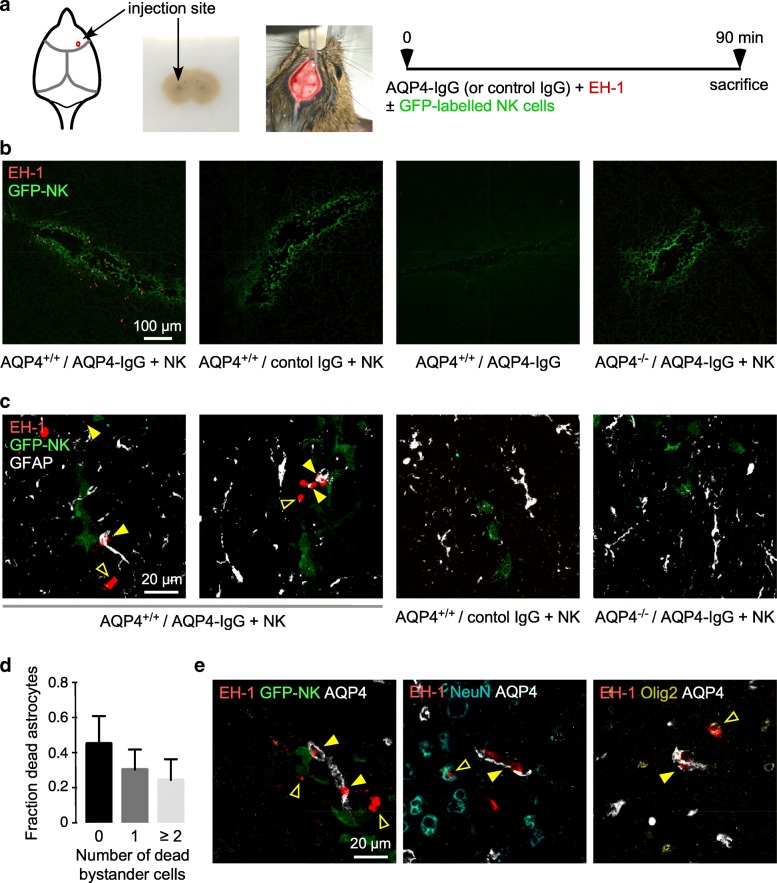
ADCC bystander killing in mouse brain. **a** Mice were administered AQP4-IgG or control IgG (4 μg) and dead cell stain EH-1 (3 μM) with or without GFP-NK cells (10^4^ cells) by intracerebral injection and sacrificed at 90 min. **b** Low-magnification confocal microscopy showing dead cells (red EH-1 fluorescence) and green immunostained NK cells. **c** High-magnification confocal images of AQP4^+/+^ or AQP4^-/-^ mice treated as in a, showing dead cells (red), GFP-NK cells (green), and astrocytes (white). Yellow filled arrows show dead astrocytes, yellow open arrows show dead bystander cells. **d** Fraction of dead astrocytes associated with 0, 1 or ≥ 2 dead bystander cells (mean ± S.E.M., 5 slides with > 30 dead cells analyzed). **e** High-magnification confocal images of mice treated as in a, with indicated stain combinations. Yellow filled arrows show dead astrocytes, yellow open arrows show dead bystander cells some of which were NeuN-positive (neurons) or Olig2-positive (oligodendrocytes)

We previously reported an ADCC model of NMO in mice in which intracerebral administration of AQP4-IgG and NK cells produced astrocytopathy and inflammation [45, 47]. Because endogenous complement activity in mouse is low [11, 46], ADCC studies in mice are not confounded by complement activation. To investigate whether ADCC bystander killing occurs in mouse brain in vivo, AQP4-IgG and a small number of GFP-labeled NK cells were injected into mouse brain together with dead cell dye ethidium homodimer-1 (EH-1) (Fig. 5a). Brains were fixed in situ and frozen sections were examined by confocal microscopy at high magnification to resolve astrocytes, NK cells and dead cells using GFAP to identify astrocytes and a GFP antibody to identify NK cells. Figure 5b shows many dead cells (EH-1 positive) near the needle tract in wild type mice receiving AQP4-IgG and GFP-NK cells. Few or no dead cells were seen with injection of control IgG and NK cells, with AQP4-IgG alone, or when AQP4-IgG and NK cells were injected into AQP4^−/−^ mice.

At high magnification, red-stained dead cells were seen, which included GFAP immunostained astrocytes (white) in contact with GFP immunostained GFP-NK cells (green), as well as some nearby dead cells that were not stained with GFAP antibody and hence not astrocytes (Figs. 5c, 2 right panels). Cell death was not seen in AQP4^+/+^ mice receiving control IgG and NK cells or AQP4^−/−^ mice receiving AQP4-IgG and NK cells (Fig. 5c, two right panels). Figure 5d summarizes the fraction of dead astrocytes associated with 0, 1 and 2 or more dead nearby (within 100 μm) bystander cells, showing dead bystander cells associated with more than half of the dead astrocytes. To identify dead bystander cells, NeuN was used as a neuron marker and Olig2 as oligodendrocyte marker, with AQP4 as the astrocyte marker. Figure 5e (left) shows bystander cell killing using AQP4 as the astrocyte marker. Figure 5e (center and right) shows examples of dead neurons and oligodendrocytes near dead astrocytes.

### Small molecule and peptide inhibitors of ADCC bystander killing

**Fig. 6:**
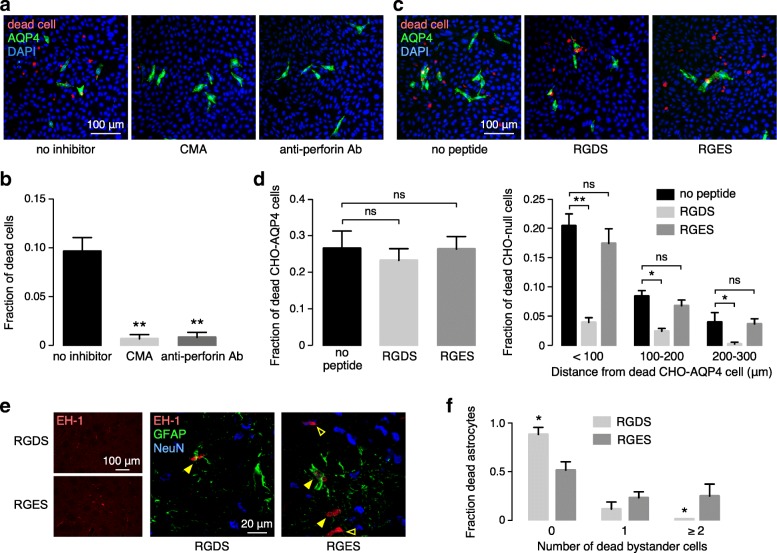
Inhibition of ADCC bystander killing by small molecule, antibody and peptide inhibitors. **a** CMA and anti-perforin antibody. Cocultures of CHO-AQP4 and CHO-null cell were incubated for 30 min with AQP4-IgG then washed and exposed for 1 h to NK cells with or without CMA (10 nM), and with or without added anti-perforin antibody (10 μg/ml), with fixable dead cell stain added 30 min prior to fixation. **b** Fraction of dead cells in cocultures from studies as in a (mean ± S.E.M., 4 slides with > 60 dead cells analyzed, ** *P*<0.01 by unpaired t test). **c** RGDS peptide. Cocultures were pre-incubated with AQP4-IgG then washed and incubated with RGDS peptide (or control RGES peptide) (200 μM) for 1 h, then exposed to NK cells with dead cell stain added 30 min prior to fixation. **d** (Left) Cultures treated as in c, showing fraction of dead CHO-AQP4 cells in cocultures with no peptide or RGDS or RGES (mean ± S.E.M., 4 slides with > 30 dead cells analyzed). (Right) Fraction of red-stained, dead CHO-null cells as a function of distance from dead CHO-AQP4 cells (mean ± S.E.M., 4 slides with > 60 dead cells analyzed, ** *P*<0.01, *P<0.05 comparing no-peptide vs. RGDS or RGES by two-way ANOVA). **e** Mice were injected with RGDS and RGES peptides (on contralateral side), together with AQP4-IgG (4 μg), NK cells (10^4^ cells) and dead cell stain EH-1 (3 μM). (Left) Low magnification showing EH-1 positive cells. (Right) High magnification confocal images showing dead cells (red), astrocytes (green) and neurons (blue). Yellow filled arrows show dead astrocytes, yellow open arrows show dead bystander cells. **f** Fraction of dead astrocytes associated with 0, 1 or ≥ 2 dead bystander cells in sections of brains from RGDS and RGES treated mice (mean ± S.E.M., 5 slides with > 30 dead cells analyzed)

We tested three potential inhibitors of ADCC in the NK cell / CHO coculture model, and one inhibitor in mice in vivo. To confirm that bystander killing in the in vitro model is perforin mediated, CHO cell cocultures were incubated with concanamycin A (CMA) or anti-perforin antibody (Fig. 6a). CMA inactivates perforin prior to release by increasing granule pH [29, 65]. Anti-perforin antibody blocks target cell killing by NK cells by a mechanism involving impaired perforin binding to the target cell surface [30]. We found near-complete prevention of target and bystander cell killing by CMA or anti-perforin antibody (Fig. 6b), supporting the conclusion that perforin is the major cytotoxic granule component in the model system studied here.

If ADCC bystander killing involves physical movement of an activated NK cell from a target CHO-AQP4 cell to a nearby bystander cell, then inhibition of NK cell adhesion to target cells might reduce bystander cell killing. NK-cell mediated ADCC can involve LFA-1/ICAM-1 interaction [7, 61], though CHO cells do not express ICAM-1 at significant levels [5, 56], nor do astrocytes under resting conditions [1, 58]. Integrin-mediated signaling may facilitate ADCC in the absence of ICAM-1 [3]. As integrin adhesion generally involves the minimal integrin binding motif Arg-Gly-Asp (RGD) [28], RGD-containing peptides can block integrin-associated cell adhesion [8, 13, 17, 50] and inhibit degranulation of cytotoxic lymphocytes [18]. CHO cells express multiple integrins, including αvβ3 and αIIbβ3, which can be blocked by RGDS peptide [14]. Figure 6c shows reduced ADCC bystander killing by Arg-Gly-Asp-Ser (RGDS) peptide preincubation, without significant effect of control peptide (Arg-Gly-Glu-Ser, RGES). The peptides did not impair killing of CHO-AQP4 target cells (Fig. 6d, left panel). Figure 6d (right panel) summarizes the fraction of dead CHO-null cells at different distances from the center of dead AQP4-expressing CHO cells, showing the RGDS preferentially reduces bystander killing within 100 μm of dead CHO-AQP4 cells.

As intracerebral injection of RGDS peptides has demonstrated utility in treating N-methyl-D-aspartate-mediated brain excitotoxicity [43], we studied RGDS action in the mice in which the peptide (or control RGES) was administered by intracerebral injection together with AQP4-IgG and NK cells. At low magnification, staining with EH-1 was much reduced with RGDS peptide compared with control RGES peptide in the contralateral hemisphere (Fig. 6e, left). Higher magnification (right panel) shows that the dead cells with RGDS peptide consisted mainly of GFAP-immunopositive astrocytes, whereas many GFAP-negative cells were killed with control peptide, some of which were NeuN-positive neurons. Quantification of images in Fig. 6f showed a significant redistribution of the cell killing profile to astrocytes vs. bystander cells with RGDS peptide.

## Discussion

The results here extend prior evidence on ADCC as a complement-independent mechanism of astrocyte injury in AQP4-IgG-seropositive NMOSD [34, 45, 47, 67, 68]. We show ADCC bystander killing as an additional pathogenesis mechanism linking AQP4-IgG binding to AQP4 on astrocytes with injury to other cell types in the central nervous system, such as neurons and oligodendrocytes, and consequent neurological deficit. ADCC bystander killing was demonstrated in AQP4-transfected CHO cell / null cell and astrocyte / neuron co-cultures using NK cells or neutrophils as the effector cells. Distance-dependent bystander cell killing and perforin immunofluorescence provided evidence in support of an ADCC bystander mechanism. The range of bystander killing in ADCC extended well beyond that seen with complement bystander injury in the same coculture system, with killing seen to 300 μm with ADCC compared to < 100 μm with CDC, demonstrating ADCC bystander injury has a greater range of action than complement bystander injury. Time-lapse imaging revealed NK cell activation upon binding to AQP4-IgG on CHO-AQP4 cells and physical movement to nearby cells, producing sequential attack of both the target and bystander cells in a contact-dependent manner. This movement of activated NK cell leads to a greater distance range for ADCC bystander killing than that for complement bystander killing. Within the distance range of ADCC bystander effect, whether a particular bystander cell is killed depends on contact with an activated NK cell with remaining cytotoxic granules and effective delivery of granule contents. Therefore, only a fraction of bystander cells are killed in a stochastic manner within the effective distance range of ADCC bystander killing.

**Fig. 7:**
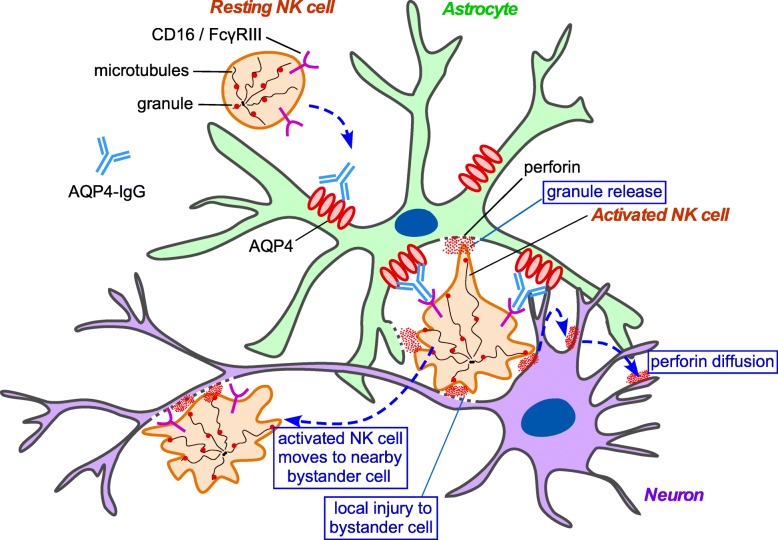
Proposed mechanisms of ADCC bystander killing in AQP4-IgG seropositive NMOSD. Diagram shows AQP4-IgG binding to AQP4 on astrocytes, with binding and activation of NK cells through Fcγ receptors. Activated NK cells can induce bystander cell damage via three mechanisms, including: (i) targeted lytic protein secretion onto immediately adjacent bystander cells following activation; (ii) sequential attack of target cells followed by release and binding/attack of a nearby bystander cell; and (iii) release of soluble granule contents following activation that then diffuse through the extracellular medium and are deposited on nearby cells

Figure 7 depicts potential mechanisms of ADCC bystander killing in NMO. AQP4-IgG binds to AQP4 on astrocytes, which allows binding and activation of NK cells through Fcγ receptors. Activated NK cells can induce bystander cell injury by several mechanisms, including: (i) targeted lytic protein secretion onto immediately adjacent bystander cells following activation; (ii) sequential attack of target cell followed by release and binding/attack of a nearby bystander cell; and (iii) release of soluble granule contents following activation that then diffuse through the extracellular medium and are deposited on nearby cells. Cytotoxic lymphocytes such as NK cells and cytotoxic T-lymphocytes store a variety of cytotoxic proteins within lytic granules, including perforin and granzyme, which are released after antigen-stimulated degranulation [59]. Target cell death relies on the diffusion of the secreted cytolytic proteins through the extracellular matrix to reach their target [48]. Perforin creates large pores on the plasma membrane of target cells, similar to membrane attack complex in complement-dependent cytotoxicity, which facilitate access of granzyme to the cytosol and subsequent cell lysis [64].

Off-target deposition of perforin by activated NK cells has been shown to injure bystander cells in other systems. Co-stimulation of Fcγ receptors (CD16) and LFA-1 was demonstrated to mediate cell specificity and reduce bystander killing of non-adherent Drosophila S2 cells [9]. In contrast, NK cells activated by CD16 alone, without LFA-1 co-stimulation, trigger bystander cell lysis by non-directional secretion of lytic granules [26]. In our experiments, NK cells were activated by AQP4-IgG coated cells by CD16 alone, as CHO cells do not endogenously express LFA-1 ligands, suggesting nonspecific release of perforin and local killing of bystander cells. However, by time-lapse imaging, we found that NK cells sequentially kill CHO-AQP4 cells followed by binding to and killing of nearby null cells, and that integrin blockade with an RGD peptide reduced bystander killing. This finding supports a modified mechanism of bystander killing in which sequential binding to bystander cells by CD16-activated NK cells causes targeted degranulation and cell death, consistent with data showing that NK cells induce serial killing at a single-cell level [15]. Other studies reported that killing of a target cell requires only one-hundredth of the total lytic granule content of an NK cell [22], which make them capable of disengaging from one target cell to serially kill additional target cells. The inhibition of cell killing by CMA and anti-perforin antibody suggests that both target and bystander cell lysis is perforin-dependent; however, it is possible that additional Fas-dependent cell killing may occur on longer time scales [2].

It remains to be determined if the ‘serial bystander’ killing described here is a general phenomenon or specific to AQP4-IgG activated NK cells. The M23 isoform of AQP4 forms large semi-crystalline macromolecular aggregates called orthogonal arrays of particles (OAPs) that are unique to this protein and that can bind several AQP4-IgG antibodies simultaneously [16, 44]. Though CDC requires AQP4 aggregation into OAPs for multivalent interaction between AQP4-IgG and complement protein C1q, ADCC is relatively insensitive to the AQP4 aggregation state [44]. Large OAPs can be removed from the membrane of cells [57], which might provide a mechanism for sustained signaling via CD16 after NK cell disengagement from an M23-expressing target cell.

Neutrophils, eosinophils and macrophages are abundantly seen in lesions in human seropositive NMOSD [33, 37, 49, 52], with NK cells and cytotoxic T-lymphocytes observed less frequently [51]. Each of these cell types express Fc receptors that can bind to the Fc moiety of AQP4-IgG. Recent studies have shown that neutrophils can mediate ADCC by mechanisms including degranulation of cytotoxic molecules [1] and releasing of neutrophil extracellular traps [12, 55], as well as inducing apoptosis [25], autophagy [6] and trogocytosis [36, 62]. We found here that neutrophils not only cause ADCC, but also ADCC bystander injury, demonstrating target non-specificity as a common feature of multiple ADCC mechanisms in NMO.

Though our results demonstrate complement-independent ADCC bystander killing in in vitro and mouse models of NMO, the study here cannot prove directly that ADCC bystander injury occurs in human seropositive NMO. In order to identify dead bystander cells in vivo, our NMO mouse model necessitated synchronizing injection of AQP4-IgG, NK cells and a dead cell marker and sacrifice at an early time point. Various leukocytes, including granulocytes and NK cells, can be short-lived after their entry into the central nervous system and activation [9], so that their paucity or absence does not rule out a role in NMO disease pathogenesis. Further, the lack of availability of central nervous system tissue during acute NMO attacks limits the extrapolation of animal data to human NMO. However, the leukocyte infiltration in necrotic lesions in post-mortem NMO samples [33, 37, 49] suggests these cells play a role in widespread tissue damage.

## Conclusions

In conclusion, our results support an ADCC bystander killing mechanism for injury to non-AQP4-expressing cells in the central nervous system following AQP4-IgG binding to astrocytes and leukocyte activation through Fcγ receptor interaction. The close proximity and intermingling of astrocytes and their processes with multiple cell types, including neurons and oligodendrocytes, would amplify bystander killing efficiency when compared to in vitro studies done on planar cell monolayers. Unlike complement bystander injury, ADCC bystander injury is independent of cell-specific expression of complement regulator proteins proteins such as CD55 and CD59, and so could produce injury to cell types that are relatively resistant to complement injury. An ADCC bystander injury mechanism would suggest the potential utility of NMO therapies directed against new targets such as perforin and leukocyte co-receptors involved in granule release, as well as adhesion molecules involved in leukocyte-bystander cell interaction.

## Additional files

Additional file 1: **Video S1.** Complement bystander killing in CHO-AQP4 and CHO-null cell coculture. Time-lapse imaging showing injured CHO-AQP4 cells (labeled with green cell tracker) and nearby CHO-null cells with uptake of dead cell marker EH-1 (red) following addition of 10 μg/ml AQP4-IgG and 5% human complement. Video includes a 30 min baseline recording with AQP4-IgG alone, with human complement added at 30 min. (AVI 3262 kb)

## Data Availability

The datasets used and/or analysed during the current study are available from the corresponding author on reasonable request.
